# Disability, Fatigue and Mental Health in Acute Versus Chronic Spinal Pain in the Gaza Strip: A Comparative Cross‐Sectional Study

**DOI:** 10.1002/msc.70274

**Published:** 2026-10-10

**Authors:** Marwa Salama, Ahmed A. Najim, Magdy Shabana, Reham Almukbel, Kinan Mokbel

**Affiliations:** ^1^ Department of Physical Therapy Faculty of Applied Medical Sciences Al‐Azhar University‐Gaza Gaza Gaza Strip Palestine; ^2^ Department of Nursing Al‐Azhar University‐Gaza Gaza Gaza Strip Palestine; ^3^ Department of Health and Care Professions Faculty of Health and Life Sciences University of Exeter Exeter UK; ^4^ German International University Cairo Egypt; ^5^ Faculty of Medicine Al‐Sham Private University Al‐Tal Syria

**Keywords:** chronic pain, fatigue, low back pain, neck pain, psychological distress, spinal pain

## Abstract

**Background:**

Spinal pain is a major contributor to disability, but evidence comparing multidomain outcomes across acute and chronic stages remains limited in conflict‐affected settings. This study aimed to compare disability, fatigue, psychological distress and sleep disturbance among patients with acute and chronic neck pain and low back pain (LBP) in the Gaza Strip.

**Methods:**

This comparative cross‐sectional study included 410 adults attending outpatient physiotherapy at Nasser Medical Complex: acute neck pain (*n* = 101), chronic neck pain (*n* = 103), acute LBP (*n* = 102) and chronic LBP (*n* = 104). A 12‐week cut‐off defined acute and chronic pain. Disability was assessed using the Neck Disability Index (NDI)/Oswestry Disability Index (ODI) scores, fatigue using the Fatigue Severity Scale (FSS), psychological distress using the Patient Health Questionnaire‐4 (PHQ‐4) and sleep disturbance using the PROMIS Sleep Disturbance Short Form 8a. Between‐group comparisons, adjusted linear regression, correlation analyses, clinical‐threshold analyses and logistic regression were performed.

**Results:**

Chronic neck pain and chronic LBP were associated with higher disability, fatigue and psychological distress than acute pain (all primary comparisons *p* < 0.001). Sleep disturbance did not significantly differ between the acute and chronic groups. Adjusted analyses showed that chronic pain status remained associated with higher disability, fatigue and psychological distress. Fatigue was independently associated with chronic pain status in both neck pain and LBP models. Female participants reported higher psychological distress in both pain‐location groups.

**Conclusions:**

Chronic spinal pain was associated with greater disability, fatigue and psychological distress but not with higher sleep disturbance. Findings support early multidimensional assessment, including screening for fatigue and psychological distress.

**Abbreviations**
BMI: Body mass index
CI: Confidence interval
FSS: Fatigue Severity Scale
LBP: Low back pain
NDI: Neck Disability Index
ODI: Oswestry Disability Index
OR: Odds ratio
PHQ‐4: Patient Health Questionnaire‐4
PROMIS: Patient‐Reported Outcomes Measurement Information System
SD: Standard deviation
SE: Standard error

## 1 Background

Low back pain (LBP) alone affected an estimated 619 million people worldwide in 2020 and is projected to affect 843 million people by 2050 (GBD 2021 Low Back Pain Collaborators 2023). Neck pain is also highly prevalent, with recent estimates showing more than 200 million prevalent cases globally and projections indicating that its population burden will remain substantial over time (GBD 2021 Neck Pain Collaborators 2024; Zhu et al. 2026). Together, LBP and neck pain are among the leading contributors to years lived with disability worldwide (Hurwitz et al. 2018).

Spinal pain is commonly classified according to symptom duration, and in this study, acute pain was defined as symptoms lasting up to 12 weeks, while chronic pain was defined as symptoms persisting beyond this period (International Association for the Study of Pain, n.d.). Although many patients recover during the acute phase, a substantial proportion develop persistent symptoms. In LBP, transition from acute to chronic pain has been reported in approximately one‐third of patients in primary care settings (Stevans et al. 2021). Neck pain can also follow persistent trajectories over time, with some patients experiencing ongoing or recurrent symptoms rather than full recovery (Irgens et al. 2022). The mechanisms underlying pain persistence are multifactorial. Sustained nociceptive input can increase excitability within central nociceptive pathways and reduce inhibitory control, producing pain hypersensitivity including hyperalgesia and allodynia (Woolf 2011). Contemporary pain phenotyping also recognises nociplastic mechanisms in some people with chronic musculoskeletal pain, which may coexist with nociceptive or neuropathic mechanisms (Nijs et al. 2021). Neuroimmune signalling, inflammatory mediators and altered excitatory–inhibitory neurotransmission have also been implicated in pain chronification (Shang et al. 2026). These mechanisms were not measured in the present study but provide a biological context for the persistence of symptoms beyond the acute stage.

Chronic spinal pain is rarely limited to pain alone. It is often accompanied by disability, fatigue, psychological distress and sleep disturbance. Disability, as framed within the International Classification of Functioning, Disability and Health, includes impairments, activity limitations and participation restrictions (World Health Organization 2002). In spinal pain, disability is clinically important because it reflects the extent to which pain affects daily functioning, work, self‐care and participation. Previous studies have identified disability as an important clinical feature in chronic neck pain (De Pauw et al. 2015) and reported differences in disability and emotional status between chronic neck pain and chronic LBP populations (Altuğ et al. 2015).

Fatigue is another important but often under‐assessed feature of chronic spinal pain. In chronic LBP, fatigue has been linked with pain intensity, depressive symptoms and disability (Snekkevik et al. 2014). Evidence from chronic musculoskeletal pain populations also suggests a temporal relationship between pain and fatigue, with earlier changes in pain severity predicting later changes in fatigue during rehabilitation (Yamada et al. 2022). This makes fatigue a clinically relevant outcome when comparing acute and chronic spinal pain groups.

Psychological distress is also central to the chronic spinal pain experience. Depression and anxiety are closely associated with persistent pain, and longitudinal evidence has reported an association between chronic LBP and subsequent depression or anxiety symptoms, although genetically informed analyses suggest that shared familial factors may partly explain this relationship (Fernandez et al. 2017). A systematic review of prognostic factors for LBP chronicity also identified psychological factors, including depression and anxiety, as important contributors to persistent pain (Nieminen et al. 2021). More broadly, clinically significant depressive and anxiety symptoms are common among adults with chronic pain (Aaron et al. 2025). These findings support the need to assess mental health alongside physical disability in patients with spinal pain.

Sleep disturbance is another relevant domain. Poor sleep may be both a consequence of pain and a factor that contributes to poorer pain outcomes. Prospective evidence suggests that sleep can act as a prognostic factor in LBP outcomes (Silva et al. 2024), and systematic‐review evidence suggests prospective associations in both directions between sleep problems and chronic musculoskeletal pain, although the bidirectional nature of this relationship requires further investigation (Runge et al. 2024). However, the relationship between sleep and spinal pain may be influenced by wider contextual factors, especially in settings affected by conflict, insecurity and displacement.

Sex differences should also be considered when studying spinal pain outcomes. Women have been reported to experience a higher prevalence of chronic neck pain and chronic LBP than men (Palacios‐Ceña et al. 2021). Broader literature on chronic musculoskeletal conditions also highlights sex‐related differences in pain prevalence, pain experience and associated outcomes (Overstreet et al. 2023). Sex‐ and gender‐related factors in LBP management may reflect a combination of biological, psychosocial and healthcare‐access factors (Bizzoca et al. 2023). Recent evidence further suggests that sex may influence the relationship between pain characteristics, disability and quality of life in people with chronic spinal pain (Van Bogaert et al. 2024).

Most previous studies have examined neck pain and LBP separately, or have focused on a single outcome such as disability, psychological distress or sleep. Fewer studies have assessed disability, fatigue and mental health outcomes simultaneously across acute and chronic stages of both neck pain and LBP. Evidence from conflict‐affected and low‐resource settings is especially limited, despite evidence that displacement, trauma and migration‐related stressors are associated with chronic pain, psychological distress and functional impairment (Nissen et al. 2022; Strømme et al. 2021; Tsetseri et al. 2024).

The Gaza Strip provides an important context for this research. Since October 2023, the ongoing conflict has severely disrupted healthcare access and intensified population‐level psychological distress. A United Nations humanitarian update citing the World Health Organization reported that the number of people in Gaza requiring mental health and psychosocial support had increased from approximately 485,000 before October 2023 to more than 1 million by 2025 (United Nations Office for the Coordination of Humanitarian Affairs 2025). In this context, patients with spinal pain may experience a compounded burden shaped by physical symptoms, psychological stress, displacement and limited access to rehabilitation.

This study therefore aimed to compare disability, fatigue and mental health outcomes, including psychological distress and sleep disturbance, among patients with acute and chronic neck pain and LBP attending a major tertiary hospital in the Gaza Strip. The study also examined sex differences in these outcomes and explored factors associated with chronic spinal pain status.

## 2 Methods

### 2.1 Study Design and Setting

A comparative cross‐sectional study was conducted from mid‐August to December 2025 in the outpatient physiotherapy department at Nasser Medical Complex, Khan Younis, Gaza Strip, Palestine. Nasser Medical Complex is one of the main government hospitals in Gaza and remained operational during the study period, providing outpatient physiotherapy services for musculoskeletal conditions, including neck pain and LBP. Because participants were assessed at a single time point after pain duration had already been established, the study did not prospectively observe transition from acute to chronic pain and cannot estimate a transition rate.

### 2.2 Participants

Adults aged 18 years or older attending outpatient physiotherapy with neck pain or LBP were eligible. Participants were classified by pain location and duration into acute neck pain, chronic neck pain, acute LBP and chronic LBP. Acute spinal pain was defined as symptoms lasting 12 weeks or less, while chronic spinal pain was defined as symptoms persisting for more than 12 weeks (International Association for the Study of Pain, n.d.).

Patients were excluded if they had autoimmune or systemic inflammatory disease, malignancy, spinal surgery within the preceding 6 months, previous traumatic spinal injury, congenital or acquired spinal defect, pregnancy, neurological disorder, severe cognitive impairment or current or previous dependence on systemic corticosteroids.

### 2.3 Sampling and Sample Size

A non‐probability consecutive sampling method was used. The target sample size was calculated using OpenEpi version 3 for estimating the frequency in a population, assuming a population size of 1,000,000, expected outcome frequency of 32%, confidence limits of 5%, design effect of 1 and confidence level of 97%. This gave a required sample size of 410 participants. The expected frequency used for sample‐size estimation was informed by previously reported transition from acute to chronic LBP (Stevans et al. 2021) and was used solely as a planning parameter rather than as an expected transition rate for the present cross‐sectional sample. The final analysed sample included 410 participants: 204 with neck pain and 206 with LBP, classified as acute neck pain (*n* = 101), chronic neck pain (*n* = 103), acute LBP (*n* = 102) and chronic LBP (*n* = 104). A 41‐participant pilot study was conducted to assess questionnaire clarity and procedural feasibility; pilot participants were not included in the final analysis.

### 2.4 Outcome Measures

Disability was assessed using context‐adapted scoring of region‐specific measures. Participants with neck pain completed the Neck Disability Index (NDI; Vernon and Mior 1991), while those with LBP completed a modified version of the Oswestry Low Back Pain Disability Questionnaire (ODI; Fritz and Irrgang 2001). The NDI driving item and ODI travelling item were retained in the administered questionnaires but excluded from the total analysed scores. During the study period, inability to drive or travel could reflect conflict‐related restrictions on vehicles, fuel, safety and routine mobility rather than pain‐related disability. Scoring these items could therefore attribute externally imposed restrictions to the spinal condition and introduce context‐related measurement error. The remaining nine items were summed as raw scores ranging from 0 to 45, with higher scores indicating greater disability. This context‐specific adaptation was intended to preserve item relevance and content validity in the study setting (Beaton et al. 2000; Terwee et al. 2018).

Fatigue was assessed using the Fatigue Severity Scale (FSS), scored from 9 to 63. A total score of 36 or above was considered clinically significant fatigue (Krupp et al. 1989). Psychological distress was assessed using the Patient Health Questionnaire‐4 (PHQ‐4), scored from 0 to 12, with higher scores indicating greater distress. Brief depression and anxiety screening measures have demonstrated utility in chronic pain and persistent musculoskeletal pain populations (Bisby et al. 2022; Vancampfort et al. 2025), and psychological outcome assessment is consistent with recommended approaches for chronic musculoskeletal pain research (Kroenke et al. 2019). Sleep disturbance was assessed using the PROMIS Sleep Disturbance Short Form 8a and analysed as a raw summed score. Internal consistency was acceptable for all instruments: Cronbach's alpha was 0.818 for NDI, 0.771 for ODI, 0.844 for FSS, 0.777 for PHQ‐4 and 0.775 for PROMIS Sleep Disturbance. All questionnaires and study instruments are provided in the Supporting Information.

### 2.5 Data Collection and Statistical Analysis

After providing written informed consent, participants completed a structured questionnaire in a researcher‐administered interview. Separate questionnaire versions were used for participants with neck pain and LBP. Sociodemographic and clinical variables included sex, age group, employment status, pain duration and previous physiotherapy for the current complaint.

Data were analysed using Stata/SE version 19 (StataCorp, College Station, TX, USA). Missing questionnaire responses were minimal, and analyses used available data for each outcome. Values outside the possible scoring range of an instrument were treated as missing. Categorical variables were summarised as frequencies and percentages, and continuous variables as mean ± standard deviation.

Independent sample *t*‐tests compared disability, fatigue, psychological distress and sleep disturbance between acute and chronic pain groups, with separate analyses for neck pain and LBP. Sex differences were also examined using independent sample *t*‐tests. Pearson correlations examined associations between sleep disturbance, fatigue, disability and psychological distress. Binary logistic regression models were conducted separately for neck pain and LBP to identify factors independently associated with chronic pain status, coded as acute pain = 0 and chronic pain = 1. PROMIS, FSS, PHQ‐4 and NDI were entered in the neck pain model; PROMIS, FSS, PHQ‐4 and ODI were entered in the LBP model.

Additional location‐specific logistic‐regression sensitivity analyses were conducted adjusting for sex, age group and employment status. Age group was entered categorically, with 18–20 years as the reference category. Previous physiotherapy was not included in these sensitivity models because no participant with acute neck pain had received previous physiotherapy, resulting in complete separation in the neck pain model; to maintain comparable model specifications across pain locations, it was omitted from both sensitivity models.

Adjusted linear regression models examined acute/chronic differences after adjustment for sex, age group, employment status and previous physiotherapy. Cohen's d was used to estimate between‐group effect sizes. Clinical‐threshold analyses used established cut‐offs for clinically significant fatigue and PHQ‐4 distress categories. Mann‐Whitney U tests and Spearman rank correlations were conducted as sensitivity analyses, and exploratory sex‐by‐pain‐status interaction models assessed whether sex modified associations between pain status and outcomes.

Regression findings were interpreted as associations rather than evidence of temporal prediction or causation. Statistical significance was set at *p* < 0.05. Primary analyses were prespecified and clinically defined; exploratory and sensitivity analyses were interpreted cautiously with attention to the number of comparisons.

### 2.6 Ethical Considerations

Ethical approval was granted by the Helsinki Committee (HC) for ethical approval by the Palestinian Health Research Council (PHRC), Gaza Strip, Palestine (approval number PHRC/HC/1240/25; 04 Aug 2025). Administrative approval was obtained from the Ministry of Health, State of Palestine (Human Resources Development Directorate; correspondence number 25114150; 13 Aug 2025). Permission to conduct the research was also obtained from Al‐Azhar University (dated: 31 July 2025). Informed consent was obtained from all participants before participation. De‐identified study data were stored securely and were accessible only to the research team.

### 2.7 Use of Large Language Models

ChatGPT was used to support manuscript language editing. The authors verified the final manuscript content and take full responsibility for the work. No large language model generated primary data or was listed as an author.

## 3 Results

### 3.1 Sample Characteristics

The study included 410 participants attending the outpatient physiotherapy department with either neck pain or LBP. Of these, 204 participants had neck pain and 206 had LBP. Using the 12‐week cut‐off, participants were classified into four groups: acute neck pain (*n* = 101), chronic neck pain (*n* = 103), acute LBP (*n* = 102) and chronic LBP (*n* = 104) (Figure 1). Overall, 203 participants had acute spinal pain and 207 had chronic spinal pain. Female participants represented 64.6% of the sample, 55.4% were unemployed, and 72.7% had not received previous physiotherapy for the current complaint (Supporting Information S1: Table S1).

**FIGURE 1:**
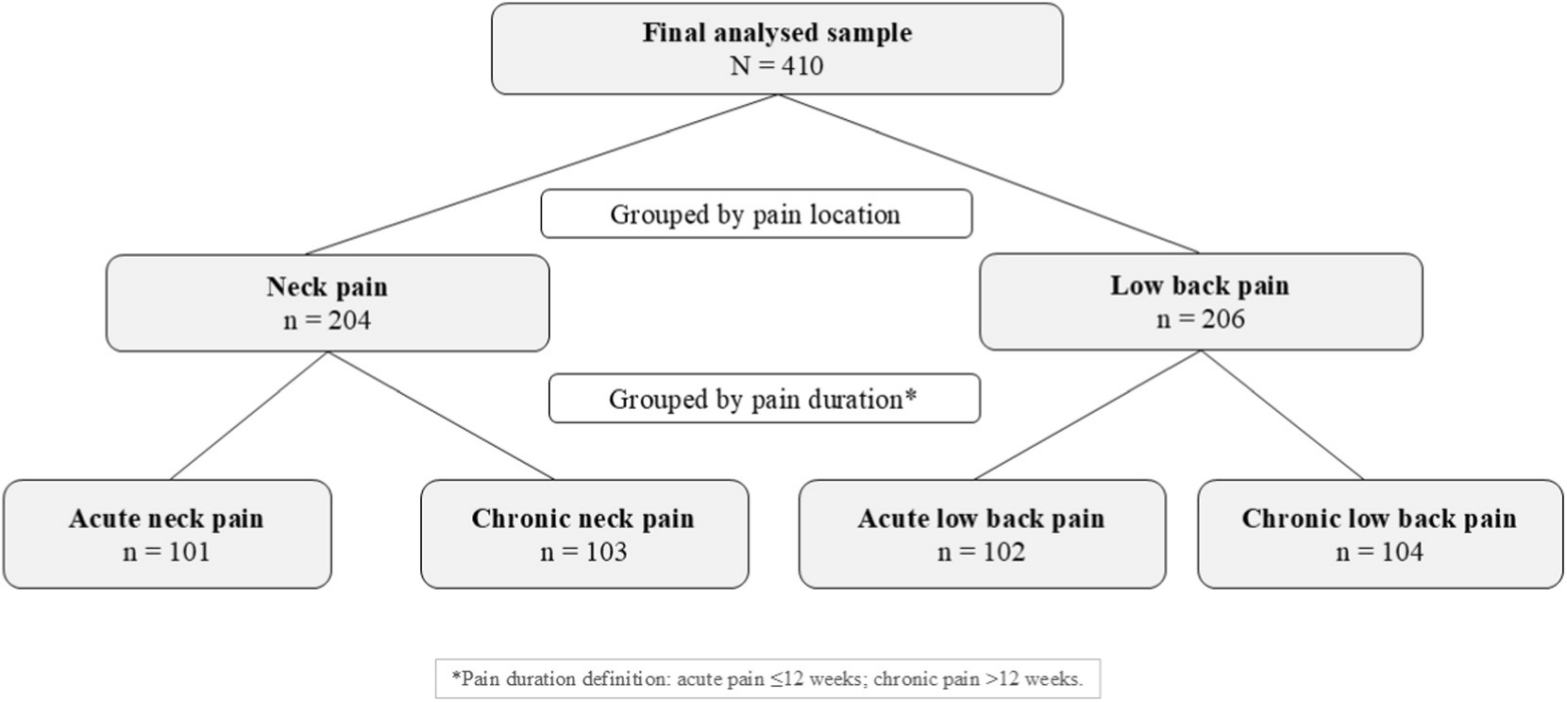
Flow chart of participant grouping by pain location and pain duration. Participants were first grouped by pain location into neck pain and low back pain groups, and then by pain duration into acute pain (≤ 12 weeks) and chronic pain (> 12 weeks).

### 3.2 Primary Outcome Comparisons

Patients with chronic spinal pain reported significantly higher disability than those with acute pain (Table 1). Participants with chronic neck pain had higher NDI scores than those with acute neck pain (21.59 ± 7.78 vs. 11.98 ± 5.46; *p* < 0.001), while participants with chronic LBP had higher ODI scores than those with acute LBP (17.29 ± 7.36 vs. 12.09 ± 5.64; *p* < 0.001). Fatigue was also significantly higher in chronic pain groups (Table 2), with both chronic pain groups exceeding the clinically significant fatigue threshold. Psychological distress followed the same pattern: chronic neck pain and chronic LBP groups had higher PHQ‐4 scores than their acute counterparts (*p* < 0.001) (Table 3). In contrast, PROMIS sleep disturbance scores did not significantly differ between acute and chronic groups for either neck pain or LBP.

**TABLE 1:** Mean differences in disability scores between acute and chronic spinal pain groups.

Outcome measure	Acute pain mean ± SD	Chronic pain mean ± SD	*t*	*p*‐value	Cohen's d
NDI, neck pain	11.98 ± 5.46 (*n* = 101)	21.59 ± 7.78 (*n* = 103)	−10.202	< 0.001	1.43
ODI, low back pain	12.09 ± 5.64 (*n* = 102)	17.29 ± 7.36 (*n* = 104)	−5.684	< 0.001	0.79

*Note:* Values are mean ± SD. NDI and ODI were analysed as adapted nine‐item raw total scores ranging from 0 to 45.

Abbreviations: NDI, Neck Disability Index; ODI, Oswestry Disability Index; SD, standard deviation.

**TABLE 2:** Mean differences in fatigue severity between acute and chronic spinal pain groups.

Outcome measure	Acute pain mean ± SD	Chronic pain mean ± SD	*t*	*p*‐value	Cohen's d
FSS, neck pain	27.63 ± 13.27 (*n* = 101)	42.10 ± 8.09 (*n* = 102)	−9.385	< 0.001	1.32
FSS, low back pain	29.13 ± 14.14 (*n* = 102)	41.89 ± 10.70 (*n* = 104)	−7.318	< 0.001	1.02

*Note:* Values are mean ± SD.

Abbreviations: FSS, Fatigue Severity Scale; SD, standard deviation.

**TABLE 3:** Mean differences in psychological distress and sleep disturbance between acute and chronic spinal pain groups.

Measure	Group	Acute pain Mean ± SD	Chronic pain Mean ± SD	*t*	*p*‐value	Cohen's d
PHQ‐4	Neck pain	3.64 ± 3.17 (*n* = 100)	6.94 ± 2.79 (*n* = 103)	−7.880	< 0.001	1.11
PHQ‐4	Low back pain	4.00 ± 3.31 (*n* = 102)	6.31 ± 3.64 (*n* = 104)	−4.761	< 0.001	0.66
PROMIS sleep disturbance	Neck pain	25.23 ± 4.87 (*n* = 100)	25.14 ± 5.73 (*n* = 102)	0.124	0.902	−0.02
PROMIS sleep disturbance	Low back pain	24.52 ± 4.84 (*n* = 102)	25.91 ± 6.95 (*n* = 104)	−1.667	0.097	0.23

*Note:* Values are mean ± SD.

Abbreviations: PHQ‐4, Patient Health Questionnaire‐4; PROMIS, PROMIS Sleep Disturbance Short Form 8a raw summed score; SD, standard deviation.

### 3.3 Sex Differences and Factors Associated With Chronic Pain Status

Sex‐based comparisons are shown in Supporting Information S1: Tables S2 and S3. Female participants reported higher psychological distress in both pain‐location groups. They also reported higher fatigue in the neck pain group (*p* < 0.001) and higher disability in the LBP group (*p* = 0.016), while sleep disturbance did not differ significantly by sex.

Binary logistic regression analyses were conducted separately for neck pain and LBP groups (Tables 4 and 5). In the neck pain model, higher FSS and NDI scores were independently associated with chronic neck pain status (OR = 1.061, 95% CI 1.022–1.102; *p* = 0.002 and OR = 1.148, 95% CI 1.079–1.220; *p* < 0.001, respectively). PROMIS sleep disturbance showed a negative association with chronic neck pain status (OR = 0.907, 95% CI 0.846–0.974; *p* = 0.007), while PHQ‐4 was not independently associated after adjustment for the other model variables (*p* = 0.112). In the LBP model, FSS was the only variable independently associated with chronic LBP status (OR = 1.057, 95% CI 1.025–1.089; *p* < 0.001); ODI approached but did not reach statistical significance (*p* = 0.066). PROMIS and PHQ‐4 were not independently associated with chronic LBP status. Because of the cross‐sectional design, these findings should be interpreted as associations with chronic pain status rather than as evidence of temporal prediction or causation.

**TABLE 4:** Binary logistic regression examining factors associated with chronic neck pain status.

Variable	B	SE	Wald	*p*‐value	OR (95% CI)
PROMIS	−0.097	0.036	7.241	0.007	0.907 (0.846–0.974)
FSS	0.060	0.019	9.666	0.002	1.061 (1.022–1.102)
PHQ‐4	0.111	0.070	2.530	0.112	1.118 (0.974–1.282)
NDI	0.138	0.031	19.348	< 0.001	1.148 (1.079–1.220)

*Note:* Model *n* = 201 complete cases. NDI was analysed as an adapted nine‐item raw total score ranging from 0 to 45. B represents the unstandardised logistic regression coefficient on the log‐odds scale.

Abbreviations: CI, confidence interval; FSS, Fatigue Severity Scale; NDI, Neck Disability Index; OR, odds ratio; PHQ‐4, Patient Health Questionnaire‐4; PROMIS, PROMIS Sleep Disturbance Short Form 8a raw summed score; SE, standard error.

**TABLE 5:** Binary logistic regression examining factors associated with chronic low back pain status.

Variable	B	SE	Wald	*p*‐value	OR (95% CI)
PROMIS	−0.030	0.029	1.051	0.305	0.971 (0.917–1.027)
FSS	0.055	0.015	12.968	< 0.001	1.057 (1.025–1.089)
PHQ‐4	0.054	0.054	1.029	0.310	1.056 (0.950–1.173)
ODI	0.056	0.031	3.377	0.066	1.058 (0.996–1.124)

*Note:* ODI was analysed as an adapted nine‐item raw total score ranging from 0 to 45. Model *n* = 206 complete cases. B represents the unstandardised logistic regression coefficient on the log‐odds scale.

Abbreviations: CI, confidence interval; FSS, Fatigue Severity Scale; ODI, Oswestry Disability Index; OR, odds ratio; PHQ‐4, Patient Health Questionnaire‐4; PROMIS, PROMIS Sleep Disturbance Short Form 8a raw summed score; SE, standard error.

### 3.4 Additional and Sensitivity Analyses

Correlation analyses showed positive associations among fatigue, disability, psychological distress and sleep disturbance in both pain‐location groups, with the strongest associations observed between fatigue, disability and psychological distress (*p* < 0.001) (Supporting Information S1: Tables S4 and S5). Adjusted linear regression models confirmed that chronic pain status remained significantly associated with higher disability, fatigue and psychological distress after adjustment for sex, age group, employment status and previous physiotherapy (Supporting Information S1: Table S6). Chronic pain status was not significantly associated with PROMIS sleep disturbance after adjustment. Additional logistic regression sensitivity models adjusting for sex, age group and employment status produced a pattern similar to the primary location‐specific models (Supporting Information S1: Table S7). In the neck pain group, higher FSS and NDI scores remained independently associated with chronic pain status, while PROMIS sleep disturbance retained an inverse association. In the LBP group, FSS remained the only outcome independently associated with chronic pain status. Sex, age group and employment status were not independently associated with chronic pain status in either model.

Clinical‐threshold analyses showed that clinically significant fatigue was more common in chronic than acute pain groups for both neck pain and LBP. Moderate or severe psychological distress was also more frequent in chronic pain groups (Supporting Information S1: Table S8). Non‐parametric sensitivity analyses supported the primary findings for disability, fatigue and psychological distress, although PROMIS sleep disturbance findings were less consistent and were therefore interpreted cautiously (Supporting Information S1: Table S9). Spearman rank correlations broadly supported the Pearson correlation findings, particularly the associations between fatigue, disability and psychological distress (Supporting Information S1: Table S10). Exploratory sex‐by‐pain‐status interaction analyses suggested possible interaction effects for fatigue and psychological distress in the neck pain group only; these findings were considered hypothesis‐generating because the study was not primarily powered for interaction testing (Supporting Information S1: Table S11).

## 4 Discussion

### 4.1 Principal Findings

This comparative cross‐sectional study found that chronic neck pain and chronic LBP were associated with significantly higher disability, fatigue and psychological distress than acute pain. Sleep disturbance showed a different pattern: it did not differ significantly between acute and chronic groups, although it was correlated with fatigue, disability and psychological distress in both pain‐location groups. In the location‐specific regression models, fatigue was the most consistent factor independently associated with chronic pain status. Neck disability was also independently associated with chronic neck pain status, whereas LBP disability approached but did not reach statistical significance.

Additional adjusted analyses were consistent with the main findings. Chronic pain status remained associated with higher disability, fatigue and psychological distress after adjustment for sex, age group, employment status and previous physiotherapy. Clinical‐threshold analyses also showed a greater burden of clinically significant fatigue and moderate or severe psychological distress in the chronic pain groups. The location‐specific logistic findings were materially unchanged after additional adjustment for sex, age group and employment status, supporting the robustness of the main associations to these measured demographic and socioeconomic characteristics. However, this adjustment does not exclude confounding effects of unmeasured conflict‐related factors such as displacement, trauma exposure or wider socioeconomic disruption.

These findings support a multidimensional interpretation of chronic spinal pain. Chronicity was not only reflected in higher disability scores but also in greater fatigue and psychological burden. This is clinically important because it suggests that patients with chronic spinal pain may require assessment beyond pain location and pain duration alone. However, because the study was cross‐sectional, the findings should be interpreted as associations with chronic pain status rather than evidence of causation or temporal prediction.

### 4.2 Disability and Chronic Spinal Pain

Disability was significantly higher in chronic neck pain and chronic LBP than in the corresponding acute groups. This indicates that persistent spinal pain is associated with greater functional limitation across both spinal regions. Similar findings have been reported in chronic back pain populations, where pain intensity, disability and depressive symptoms were positively correlated (Garbi et al. 2014). The present study adds to this evidence by showing that higher disability is evident when acute and chronic groups are compared directly within the same clinical setting.

Pain severity may also contribute to these differences. In patients with cervical radiculopathy, greater pain intensity has been associated with higher disability and depressive symptoms (Ferat and Apaydin 2025), while research in non‐surgical spinal pathology has linked pain severity with pain beliefs and emotion‐regulation strategies (Koç Apaydın et al. 2024). Pain intensity has also been associated with fatigue in chronic LBP (Snekkevik et al. 2014). Because the present study did not include a separate standalone measure of pain intensity, it is not possible to determine how much of the observed differences in disability, fatigue and psychological distress between acute and chronic groups was attributable to pain severity itself.

In the adjusted models, disability remained independently associated with chronic pain status in the neck pain group, while ODI approached but did not reach statistical significance in the LBP group. This suggests that disability was clearly higher in chronic pain groups, but its independent association with chronicity differed by spinal region after adjustment for fatigue, psychological distress and sleep disturbance.

Disability in spinal pain is unlikely to be explained by pain duration alone. It is more likely to reflect the interaction between persistent pain, reduced activity, emotional distress and avoidance of movement. Previous research has shown that catastrophising, fear of movement, anxiety and depression are associated with persistent severe LBP and disability (Ranger et al. 2020). A systematic review of mediation studies also suggested that psychological distress and fear may partly explain the pathway between pain and disability in musculoskeletal pain (Lee et al. 2015). This supports the interpretation that disability in chronic spinal pain is a biopsychosocial outcome rather than a purely physical consequence.

The findings are also consistent with research in non‐specific LBP showing that disability, fatigue and psychological factors vary according to the degree of chronification (Adnan et al. 2020). In the current study, a similar multidomain pattern was visible across both neck pain and LBP. Clinically, this suggests that disability screening should be included early in the assessment of spinal pain, especially when symptoms persist beyond the acute stage.

### 4.3 Fatigue as a Central Feature of Chronic Spinal Pain

Fatigue was the most consistent finding across the primary and additional analyses. It differed significantly between acute and chronic groups, remained significant in the adjusted linear models, and was the only factor independently associated with chronic pain status in both location‐specific logistic regression models. Patients with chronic neck pain and chronic LBP had substantially higher FSS scores than patients with acute pain. Both chronic pain groups also had mean fatigue scores above the clinically significant threshold, suggesting that fatigue is not a minor accompanying symptom but a central feature of the chronic spinal pain burden.

This finding is supported by previous evidence showing that patients with chronic LBP and chronic neck pain report elevated fatigue compared with non‐pain comparison groups (Fishbain et al. 2004). In neck pain specifically, fatigue may also affect neuromuscular control. Hsu et al. (2020) reported that fatigue altered neck muscle control and worsened postural stability during arm movement perturbations in patients with chronic neck pain. This provides a plausible mechanism through which fatigue may contribute to functional limitation in chronic neck pain.

In the present study, fatigue was strongly correlated with disability and psychological distress in both pain‐location groups. This is clinically important. Fatigue may reduce engagement with rehabilitation, limit activity tolerance and increase perceived disability. It may also interact with psychological distress, creating a cycle in which persistent pain, low energy, reduced activity and emotional symptoms reinforce one another. Routine fatigue screening may therefore help identify patients who need closer follow‐up, pacing advice and broader rehabilitation support.

### 4.4 Psychological Distress and Chronic Spinal Pain

Psychological distress was significantly higher in chronic pain groups than in acute pain groups. Patients with chronic neck pain and chronic LBP both reported higher PHQ‐4 scores than those with acute pain. This supports the view that chronic spinal pain is associated with a greater emotional burden.

However, PHQ‐4 did not remain independently associated with chronic pain status in the adjusted location‐specific regression models. Psychological distress should therefore be interpreted as an important marker of clinical burden rather than an independent explanatory factor in these models.

The neck pain findings are consistent with previous work showing that psychological states are associated with pain and disability among patients with chronic neck pain (Dimitriadis et al. 2015). High levels of anxiety and depression have also been reported among patients with chronic neck pain, highlighting the importance of psychological assessment in this population (Elbinoune et al. 2016). In the current study, psychological distress was strongly correlated with disability and fatigue, suggesting that emotional symptoms are closely connected to the broader clinical burden of chronic spinal pain.

However, psychological distress should not be interpreted simply as a consequence of spinal pain. In Gaza, ongoing conflict, displacement, insecurity, bereavement and restricted access to care may contribute to psychological distress across the whole population. This may partly explain why psychological distress was also present in acute pain groups. A more cautious interpretation is that chronic spinal pain status was associated with higher psychological distress within an already highly stressed context.

### 4.5 Conflict‐Related Stress, Trauma and Pain Persistence

The conflict context may have contributed to the clinical burden observed in this sample, although conflict‐related exposures were not measured directly. Displacement, trauma exposure, bereavement, socioeconomic disruption and restricted access to care may influence psychological distress, sleep, physical activity and pain‐related coping. Evidence from conflict‐affected and low‐resource settings is especially limited. Studies of Syrian refugees have reported substantial co‐occurrence of chronic pain and mental health problems, with functional impairment documented among refugees with chronic pain (Nissen et al. 2022; Strømme et al. 2021). A broader scoping review has also documented a substantial burden of chronic musculoskeletal pain among migrants from North Africa and the Middle East living in Europe (Tsetseri et al. 2024).

PTSD was not assessed in the present study, and the PHQ‐4 measures depressive and anxiety symptoms rather than PTSD. It is therefore not possible to determine whether PTSD contributed to chronic pain status, sleep disturbance or the observed sex differences, nor to examine relationships between PTSD and quality of life. Nevertheless, the recognised co‐occurrence of PTSD and chronic pain in trauma‐affected populations suggests that trauma‐related symptoms warrant consideration in future research and, where clinically appropriate, in rehabilitation assessment in conflict‐affected settings (Rometsch‐Ogioun El Sount et al. 2019).

Employment status was the socioeconomic indicator available in the dataset and was not independently associated with chronic pain status in the additional sensitivity analyses. However, employment status provides only a limited indication of socioeconomic adversity and cannot capture displacement, loss of income, housing insecurity or disrupted access to essential services. These unmeasured contextual factors may therefore have influenced the observed associations.

Prospective primary‐care cohorts in non‐conflict settings have reported transition from acute to chronic LBP in approximately one‐third of patients (Stevans et al. 2021). This estimate cannot be compared directly with the proportion of participants classified as chronic in the present study because participants were assessed cross‐sectionally rather than followed prospectively from an acute episode. Therefore, the present study cannot determine whether exposure to conflict increased the probability of transition from acute to chronic pain.

### 4.6 Sleep Disturbance Across Acute and Chronic Groups

Sleep disturbance did not significantly differ between the acute and chronic pain groups. This differs from previous evidence linking sleep disturbance with chronic spinal pain (Van Looveren et al. 2021). Systematic review and meta‐analysis evidence also supports a bidirectional relationship between chronic musculoskeletal pain and sleep‐related problems (Santos et al. 2023), while cohort data in LBP suggest that poorer sleep and chronic pain may influence one another over time (Campanini et al. 2022).

The absence of a significant between‐group difference in the present study does not imply that sleep disturbance was clinically unimportant. PROMIS sleep disturbance scores were significantly correlated with fatigue, disability and psychological distress in both neck pain and LBP groups. In the neck pain model, the inverse association between PROMIS score and chronic pain status should be interpreted cautiously because no significant difference in sleep disturbance was observed between acute and chronic neck pain groups in the unadjusted analysis. This pattern may reflect adjustment effects or shared contextual sleep disruption rather than a meaningful protective association.

The conflict setting may be particularly relevant to this finding. Sleep in Gaza may be disrupted by insecurity, displacement, overcrowding, environmental noise and psychological stress, potentially reducing differences attributable specifically to pain duration. However, displacement status, living conditions and trauma‐related sleep symptoms were not measured, so this explanation cannot be tested directly. Sleep should therefore remain part of clinical assessment, but its interpretation in conflict‐affected settings should consider both pain‐related and wider contextual influences.

### 4.7 Sex Differences in Disability, Fatigue and Psychological Distress

Female participants experienced a greater burden in several domains. In the neck pain group, female participants reported higher fatigue and psychological distress than male participants. In the LBP group, female participants reported higher disability and psychological distress than male participants. These findings are partly consistent with previous chronic LBP research, where anxiety and depression were more common among female patients (Sagheer et al. 2013).

The findings also align with broader evidence that chronic pain places a disproportionate burden on women (Barreto et al. 2025). In the current study, this burden was not identical across pain locations. In neck pain, the main sex differences were fatigue and psychological distress. In LBP, the main differences were disability and psychological distress. This suggests that sex‐sensitive assessment should consider both pain location and outcome domain.

In the Gaza context, these sex differences may also reflect unmeasured trauma‐related and socioeconomic influences. PTSD symptoms, displacement status, caregiving burden and household stress were not assessed; therefore, the relative contributions of biological, trauma‐related and socioeconomic factors cannot be determined from these data.

The exploratory interaction analyses suggested that sex may modify the association between pain status and both fatigue and psychological distress in the neck pain group. However, these analyses were exploratory and the study was not primarily powered to detect interaction effects. They should therefore be interpreted as hypothesis‐generating rather than evidence of a sex‐specific causal pathway.

### 4.8 Clinical Implications

These findings support early multidimensional assessment of patients presenting with spinal pain. Disability measures remain important, but this study suggests that fatigue and psychological distress should also be assessed routinely, particularly when symptoms persist beyond the acute stage. Fatigue was the most consistent factor associated with chronic pain status across the separate regression models, making it a potentially useful clinical marker.

The findings also support a biopsychosocial approach to spinal pain management. Physiotherapy that focuses only on local spinal symptoms may be insufficient for patients with chronic pain, high fatigue or psychological distress. Evidence from multidisciplinary chronic LBP management indicates that anxiety and depression can influence treatment outcomes (Oliveira et al. 2019). Sleep should also remain a part of the assessment as poor sleep quality has been associated with chronic neck pain (Andreucci et al. 2020).

PROMIS sleep short forms have demonstrated reliability and responsiveness in chronic pain research, although not specifically in spinal pain populations (Chimenti et al. 2021). The PHQ‐4 also has strong psychometric support as a brief measure of anxiety and depression symptoms in general adult populations (Kazlauskas et al. 2023). These instruments are practical and can help identify patients who may need additional support, closer follow‐up or referral where services are available.

In Gaza and similar settings, these recommendations need to be realistic. Rehabilitation services may be disrupted, and access to specialist mental health support may be limited. Nevertheless, simple screening for disability, fatigue, psychological distress and sleep problems may help clinicians identify patients with greater multidimensional clinical burden and tailor care accordingly.

### 4.9 Strengths and Limitations

This study has several strengths. It included a relatively large sample of 410 participants with balanced acute and chronic subgroups for both neck pain and LBP. It also assessed several clinically relevant domains at the same time, including disability, fatigue, psychological distress and sleep disturbance. The use of established outcome measures strengthens the clinical relevance of the findings. Another important strength is the setting, as evidence from Gaza and similar conflict‐affected contexts remains limited.

Several limitations should also be acknowledged. First, the cross‐sectional design prevents causal inference. The regression findings therefore identify factors associated with chronic pain status but do not establish temporal prediction or causation. Second, the study was conducted at a single centre, which may limit its generalisability. Third, a separate standalone measure of pain intensity was not collected. Although the NDI and ODI each include a pain‐intensity item, these items form part of their composite disability scores and cannot provide an independent pain‐severity covariate. This is important because pain intensity has been associated with disability and depressive symptoms in spinal pain populations (Ferat and Apaydin 2025) and with fatigue in chronic LBP (Snekkevik et al. 2014). Residual confounding by pain severity may therefore have contributed to the observed differences in disability, fatigue and psychological distress. Other potentially relevant confounders, including BMI, medication use, physical activity, smoking status and trauma exposure, were also not systematically measured. Fourth, the NDI and ODI were adapted by excluding one contextually non‐applicable item from each scale: the NDI driving item and the ODI travelling item. This was intended to reduce potential measurement error because inability to drive or travel during the conflict could reflect restricted access to vehicles, fuel, safety and routine mobility rather than pain‐related disability. However, because the NDI and modified ODI are ten‐item measures in their published forms (Vernon and Mior 1991; Fritz and Irrgang 2001), and adaptation of self‐report measures should consider experiential relevance and content validity (Beaton et al. 2000; Terwee et al. 2018), this context‐specific scoring adaptation may limit direct comparison with studies using the complete instruments. Finally, the study included several secondary and exploratory analyses. These findings, particularly sex‐by‐pain‐status interactions and sensitivity analyses, should be interpreted cautiously because formal correction for multiple testing was not applied across all comparisons.

The conflict context is both a strength and a limitation. Displacement status, trauma exposure, bereavement, household economic disruption and PTSD symptoms were not measured directly. Employment status was included as a measured socioeconomic characteristic and as a covariate in the adjusted analyses, but it cannot capture the wider socioeconomic instability created by conflict. Residual confounding by these unmeasured contextual factors therefore cannot be excluded. Consecutive sampling may also have introduced selection bias because the sample comprised patients able to access outpatient physiotherapy during a period of severe healthcare disruption.

### 4.10 Future Research

Future prospective cohort studies beginning in the acute phase are needed to estimate transition rates and examine whether pain intensity, trauma exposure, PTSD symptoms, displacement and socioeconomic disruption predict or are associated with subsequent pain persistence in conflict‐affected settings. Where feasible, inclusion of comparable populations from non‐conflict settings would help determine whether patterns of progression to persistent pain differ according to conflict exposure and context. Mixed‐method studies incorporating validated trauma and PTSD measures alongside qualitative interviews on personal trauma, emotional distress, sleep disruption, loneliness and social isolation, and barriers to rehabilitation could provide complementary insight into how these experiences interact with disability, fatigue and psychological distress. Other relevant factors, including medication use, physical activity and BMI, should also be considered.

## 5 Conclusions

This comparative cross‐sectional study found that chronic neck pain and chronic LBP were associated with significantly higher disability, fatigue and psychological distress than acute pain. Fatigue showed the most consistent association with chronic pain status across both spinal regions, while disability was higher in chronic pain groups and remained independently associated with chronicity in the neck pain model. Psychological distress was also higher in chronic pain groups, although it did not remain independently associated with chronic pain status after adjustment in the location‐specific regression models. Sleep disturbance did not significantly differ between acute and chronic groups, although it was correlated with fatigue, disability and psychological distress.

These findings support the need for early multidimensional assessment of patients presenting with spinal pain, including routine screening for fatigue, functional limitation and psychological distress. This may be particularly important in conflict‐affected and low‐resource settings, where displacement, insecurity and limited access to rehabilitation may intensify both physical and emotional burden. However, because the study was cross‐sectional, the findings should be interpreted as associations rather than evidence that these factors cause or predict chronicity. Longitudinal and multi‐centre studies are needed to clarify temporal relationships and to evaluate whether early biopsychosocial interventions can reduce progression from acute to chronic spinal pain.

## Author Contributions

**Marwa Salama:** conceptualization, investigation, data curation, formal analysis, writing – original draft, writing – review and editing. **Ahmed A. Najim:** conceptualization, methodology, writing – review and editing. **Magdy Shabana:** conceptualization, methodology, writing – review and editing. **Reham Almukbel:** formal analysis, validation, writing – review and editing. **Kinan Mokbel:** supervision, project administration, methodology, writing – original draft, writing – review and editing. All authors approved the final version of the manuscript and agree to be accountable for all aspects of the work.

## Funding

The authors have nothing to report.

## Ethics Statement

Ethical approval was granted by the Helsinki Committee (HC) for ethical approval by the Palestinian Health Research Council (PHRC), Gaza Strip, Palestine (approval number PHRC/HC/1240/25; 04 Aug 2025). Administrative approval was obtained from the Ministry of Health, State of Palestine (Human Resources Development Directorate; correspondence number 25114150; 13 Aug 2025). Permission to conduct the research was also obtained from Al‐Azhar University (dated: 31 July 2025). Informed consent was obtained from all participants before participation. De‐identified study data were stored securely and were accessible only to the research team.

## Consent

The authors have nothing to report.

## Conflicts of Interest

The authors declare no conflicts of interest.

## Supporting information


Supporting Information S1

## Data Availability

The datasets generated and analysed during the current study are not publicly available because public sharing was not included in the ethical approval and because the dataset contains sensitive clinical information from a conflict‐affected setting. De‐identified data are available from the corresponding author on reasonable request, subject to ethical and institutional approvals.
